# Supplementary material for: Exploring Risk Factors of Recall-Associated Foodborne Disease Outbreaks in the United States, 2009–2019
Source: Int J Environ Res Public Health. 2022 Apr 19;19(9):4947. doi: 10.3390/ijerph19094947 (PMC9099668; doi:10.3390/ijerph19094947)
Supplement: Supplementary file 1 [file ijerph-19-04947-s001.zip › ijerph-1600629-supplementary.pdf]

Epidemiology and Infection

Supplementary Materials

Exploring risk factors of recall-associated foodborne disease outbreaks in the United States, 2009–2019

Emily Sanchez[1,2*], Ryan B. Simpson[1], Yutong Zhang[1], Lauren E. Sallade[1], Elena N. Naumova[1*]

1 Tufts University Friedman School of Nutrition Science and Policy, Boston, MA, USA 02111; Emily.Sanchez@tufts.edu; Ryan.Simpson@tufts.edu; Yutong.Zhang@tufts.edu; Lauren.Sallade@tufts.edu; Elena.Naumova@tufts.edu

2 Army Medical Department Student Detachment, U.S. Army Medical Center of Excellence, Fort Sam Houston, TX; Emily.Sanchez@tufts.edu

***Corresponding Authors:**

Emily Sanchez
Emily.sanchez@tufts.edu
150 Harrison Avenue, Boston, MA 02111

Elena N. Naumova
Elena.Naumova@tufts.edu
150 Harrison Avenue, Boston, MA 02111

Word Count: 844 words

Epidemiology and Infection

Supplementary Materials

Exploring risk factors of recall-associated foodborne disease outbreaks in the United States, 2009–2019

Supplementary Table S1. Summary of model coefficients and diagnostics estimated using segmented negative binomial regression analyses adjusted for linear, quadratic, and harmonic trends. We examined temporal patterns of foodborne and waterborne outbreaks, recalls, non-recalls, and outbreaks with missing recall information as reported by the electronic Foodborne Outbreak Reporting System (eFORS) and National Outbreak Reporting System (NORS) in 1998-2019. We defined 3 critical periods according to 2 critical points, including November 2004 (when CDC began food recall reporting) and January 2009 (transition from eFORS to NORS surveillance systems). We report exponentiated coefficients with standard error values and Akaike's Information Criterion (AIC) for each model.

AIC	Model	Period	Intercept	Linear Trend	Quadratic Trend	Sine Term	Cosine Term
**Outbreaks**							
2,294.212	1	1	112.669 ± 0.044[a]	0.999 ± 0.001			
	1	2		0.993 ± 0.002[a]			
	1	3		1.008 ± 0.001[a]			
2,282.219	2	1	106.928 ± 0.068[a]	1.004 ± 0.004	1.000 ± <0.000		
	2	2		1.021 ± 0.008[b]	1.000 ± <0.000[a]		
	2	3		1.027 ± 0.006[a]	1.000 ± <0.000[a]		
2,247.089	3	1	104.391 ± 0.077[a]	1.004 ± 0.006	1.000 ± <0.000[c]	1.079 ± 0.028[b]	0.923 ± 0.029[b]
	3	2		1.023 ± 0.015[b]	1.000 ± <0.000[a]	1.035 ± 0.047	1.027 ± 0.044
	3	3		1.028 ± 0.013[a]	1.000 ± <0.000[a]	1.060 ± 0.044	0.979 ± 0.042
**Recalls**							
622.1635	1	1	0.002 ± 1.492[a]	1.067 ± 0.019[a]			
	1	2		0.963 ± 0.023			
	1	3		0.972 ± 0.007[a]			
613.294	2	1	0.112 ± 1.339	0.897 ± 0.079	1.001 ± 0.001		
	2	2		0.973 ± 0.083	0.997 ± 0.001[b]		
	2	3		1.026 ± 0.026	1.001 ± <0.000[c]		
615.302	3	1	0.033 ± 1.406[c]	0.838 ± 0.097	1.002 + 0.001[c]	1.465 ± 0.651	0.233 ± 0.664[c]
	3	2		0.921 ± 0.102	0.996 ± 0.001[a]	2.545 ± 0.764	2.698 ± 0.577
	3	3		1.032 ± 0.030	1.002 ± 0.001[b]	1.192 ± 0.197	1.207 ± 0.175
**Non-Recalls**							
2273.547	1	1	3.558 ± 0.130[a]	1.026 ± 0.002[a]			
	1	2		0.995 ± 0.004			
	1	3		0.976 ± 0.003[a]			
2,030.867	2	1	8.343 ± 0.141[a]	0.982 ± 0.007[c]	1.000 ± <0.000[a]		
	2	2		1.104 ± 0.012[a]	0.997 ± <0.000[a]		
	2	3		1.093 ± 0.008[a]	1.002 ± <0.000[a]		
1,967.82	3	1	11.707 ± 0.152[a]	0.987 ± 0.007	1.000 ± <0.000[c]	0.730 ± 0.06	0.871 ± 0.062[c]
	3	2		1.099 ± 0.011	0.998 ± <0.000[b]	1.338 ± 0.077	0.669 ± 0.071
	3	3		1.083 ± 0.007	1.002 ± <0.000[c]	0.953 ± 0.052	0.999 ± 0.048
**Outbreaks Missing Recall Information**							
1,882.578	1	1	138 ± 0.132[a]	0.990 ± 0.002[a]			
	1	2		0.942 ± 0.005[a]			
	1	3		1.089 ± 0.005[a]			
1,649.601	2	1	111.239 ± 0.095[a]	0.994 ± 0.005	1.000 ± <0.000		
	2	2		0.743 ± 0.017[a]	1.004 ± <0.000[a]		
	2	3		0.873 ± 0.018[a]	0.996 ± <0.000[a]		
1,631.094	3	1	137.277 ± 0.136[a]	0.998 ± 0.005	1.000 ± <0.000	1.063 ± 0.038	0.960 ± 0.039
	3	2		0.719 ± 0.021[a]	1.005 ± <0.000[a]	1.004 ± 0.115	0.697 ± 0.104[a]
	3	3		0.843 ± 0.022[a]	0.995 ± <0.000[a]	0.804 ± 0.112	1.105 ± 0.127

Superscripts indicate statistical significance at $p<0.001$ ([a]), $p<0.01$ ([b]), and $p<0.05$ ([c]).

Supplementary Table S2. Estimated trend contribution of linear and quadratic trend using Model 6 for outbreaks, recalls, non-recalls, and outbreaks missing recall information as reported by the electronic Foodborne Outbreak Reporting System (eFORS) and National Outbreak Reporting System (NORS) in 1998-2019. We defined 3 critical periods according to 2 critical points, including November 2004 (when CDC began food recall reporting) and January 2009 (transition from eFORS to NORS surveillance systems).

Model	Period 1	Period 2	Period 3
**Outbreaks**			
Linear	0.072	0.404	0.509
Quadratic	0.001	0.006	0.007
**Recalls**			
Linear	0.649	0.166	0.152
Quadratic	0.008	0.016	0.008
**Non-Recalls**			
Linear	0.085	0.468	0.423
Quadratic	0.001	0.012	0.010
**Outbreaks Missing Recall Information**			
Linear	0.013	0.665	0.302
Quadratic	<0.001	0.010	0.010

Supplementary Table S3. Logistic regression results examining the likelihood of foodborne and waterborne outbreaks resulting in food recalls as reported by the electronic Foodborne Outbreak Reporting System (eFORS) and National Outbreak Reporting System (NORS) in 1998-2019. We selected risk factors according to univariate logistic regression results and added factors in a stepwise order. Risk factors include multistate outbreaks (Reference: single-state outbreaks), supply chain contamination stage (Reference: before preparation), and IFSAC Level 1 food categories (Reference: land animals). We report the odds ratio (with 95% confidence intervals), Akaike's Information Criterion (AIC), and the number of observations per model.

**Risk Factors**	Model 5.1	Model 5.2	Model 5.3	Model 5.4
Residence of Ill Persons				
Multistate	12.80 [8.67, 19.10][a]	28.60 [21.20, 38.90][a]		12.10 [7.83, 19.00][a]
Supply Chain Contamination Stage				
Preparation	0.09 [0.03, 0.21][a]		0.03 [0.01, 0.07][a]	0.06 [0.02, 0.16][a]
Unknown	0.53 [0.31, 0.89][c]		0.67 [0.41, 1.07]	0.48 [0.27, 0.80][c]
IFSAC Level 1				
Aquatic Animals		1.25 [0.87, 1.78]	0.40 [0.26, 0.61][a]	0.60 [0.38, 096][c]
Plants		0.78 [0.56, 1.09]	1.53 [1.03, 2.29][c]	0.76 [0.47, 1.22]
Other Foods		1.19 [0.44, 2.64]	3.25 [0.68, 12.10]	3.85 [0.77, 14.70]
Modeling Diagnostics				
AIC	812.48	1,667.67	938.94	810.20
Observations	1,738	5,691	1,738	1,738

Superscripts indicate statistical significance at p<0.001 ([a]), p<0.01 ([b]), and p<0.05 ([c]).
